# Lead isotopes of prehistoric copper tools define metallurgical phases in Late Neolithic and Eneolithic Italy

**DOI:** 10.1038/s41598-024-54825-z

**Published:** 2024-02-21

**Authors:** Gilberto Artioli, Ivana Angelini, Caterina Canovaro, Günther Kaufmann, Igor Maria Villa

**Affiliations:** 1 Dipartimento di Geoscienze, Università di Padova, via Gradenigo 6, 35131 Padova, Italy; 2 Department of Cultural Heritage: Archaeology and History of Art, Cinema and Music, University of Padua, Padova, Italy; 3 Museo Archeologico dell’Alto Adige/Südtiroler Archäologiemuseum, Bolzano/Bozen, Italy; 4 Institut für Geologie, Universität Bern, Bern, Switzerland

**Keywords:** Copper Age, Copper metal provenance, Alpine region, Northern Italy, Eneolithic, Archaeology, Geochemistry

## Abstract

The diffuse presence of small copper ore deposits in the Alpine area, mostly exploited since Late Medieval times, led most scholars to assume that these deposits may actually be active much earlier and that many of the circulating prehistoric metal objects found in the area were produced with local copper sources. This assumption was recently validated for the Recent Bronze Age through the use of lead isotope tracers, and well supported by the archaeometallurgical evidences found in the South-Eastern Alps. However, the scarcity of available lead isotope data for pre-Bronze Age metals precluded to date the reconstruction of the metal flow through the Late Neolithic and Eneolithic (or Copper Age). Based on 49 new analyses of important archaeological objects from the Alpine region, the Po River Valley and Central Italy, mostly axes dated from the Late Neolithic to the Late Eneolithic, here we show that the diffusion of copper in Northern Italy (approximately 4500–2200 BC) includes three major periods of metal use and/or production, each related to specific ore sources. The South Alpine copper was massively used only starting from the middle of the 3rd millennium BC, in connection or slightly earlier than the Beaker event.

## Introduction

The sequence of metal introduction in different areas of the world generally follows a simple pattern, commonly defined since the early nineteenth century as the “Three Age System”^1^, mostly applicable to Europe and the Near East. The introduced chronological succession of Stone Age, Bronze Age, and Iron Age was based on the seemingly universal sequence of the named materials for the manufacturing of everyday and warfare tools. However, it was soon realized that the sequence and mode of metal adoption, the relation with the cultural context, and especially the timing of metal introduction followed rather different local patterns^2,3^. Especially problematic is the archaeological identification and precise chronological definition of the early introduction and technological adoption of copper, which in many areas mark the beginning of the use of metals and precede the widespread use of alloyed copper, mostly in the form of arsenic- or tin-alloys (i.e. bronze). This copper-based phase of metallurgy, commonly labelled Copper Age (or Eneolithic or Chalcolithic), but actually starting in the Late Neolithic, is often hard to be univocally defined^4^, being sometimes evidenced by the slow adoption of very simple tools and ornaments (awls, pins, small sheets, beads) such as in Northern Italy and Central Europe^5^, and at times displaying a sudden introduction of a massive amount of metal in the form of elaborated implements (scepters/standards, axes, chisels, maces) such as in the Ghassulian culture of the Levant^6^. The understanding of the cultural and social aspects of metal adoption therefore needs the careful evaluation of at least three mutually interacting factors: (1) the geological availability of the ores through direct mining and/or the availability of the raw metal through commercial trade, (2) the technological mastering of ore smelting and/or tools manufacturing by secondary metallurgy, and (3) the cultural, social or practical need of metal implements. The evidence of locally produced metal is therefore the fundamental piece of information required to unambiguously assess the metal technology and production in any given culture.

Metal provenance is commonly assessed through the combination of chemical and isotopic tracers^7^, though the reliability of the provenance assignment critically relies on the availability of extensive reference databases of the trace parameters measured on the ore deposits. Despite earlier debates on the significance and interpretation of the lead isotope analysis (LIA) of Pb traces present in the material extracted from copper-based alloys, the recent development of robust geologically-reasoned lead isotope databases of copper ores^8,9^ granted confidence in the interpretation of the LIA data measured on copper and bronze artefacts. Quantitative chemical analyses (major, minor and trace elements) and isotopic data are nowadays systematically measured and reported for archaeological metal objects, and the growing body of data is finally allowing to define regional and trans-regional networks of metal production and exchanges, thus providing solid basis for further speculation on metal use and economy in a given area.

### Prehistoric Alpine copper production: the context

Archaeometallurgy scholars commonly assume that (1) the copper Alpine deposits were exploited since the dawn of the Copper Age, possibly in connection with the Pfyn-Altheim-Mondsee phase of copper circulation North of the Alps, (2) most of the ancient mining evidences were erased by subsequent works, starting with the systematic exploitation of ore resources in the Middle Age and peaking with massive metal production during the industrial revolution. The exploitation of Alpine ores was thoroughly validated for the Recent Bronze Age by the application of lead isotope analysis (LIA) linking ores to artefacts^10^, and archaeologically well supported by the widespread occurrence of slags and excavated smelting sites in the South-Eastern Alps^11–13^. However, for earlier periods the scarce available lead isotope data^14,15^ indicate that different ore sources were used to produce copper, so that it is unclear when exactly the Alpine ores started to be actively exploited, despite some clear archaeological evidence indicating pre-Bronze Age metallurgical activity^16,17^.

Here we address the long-standing problem of prehistoric copper production in the Alpine area and nearby regions, by analysing a number of archaeologically significant objects from the Alpine region, the Po River Valley and Central Italy. The objective is to assess the provenance of the metal composing Eneolithic artefacts used in Northern and Central Italy, to test the potential production of copper from South-Eastern Alpine deposits.

**Figure 1:**
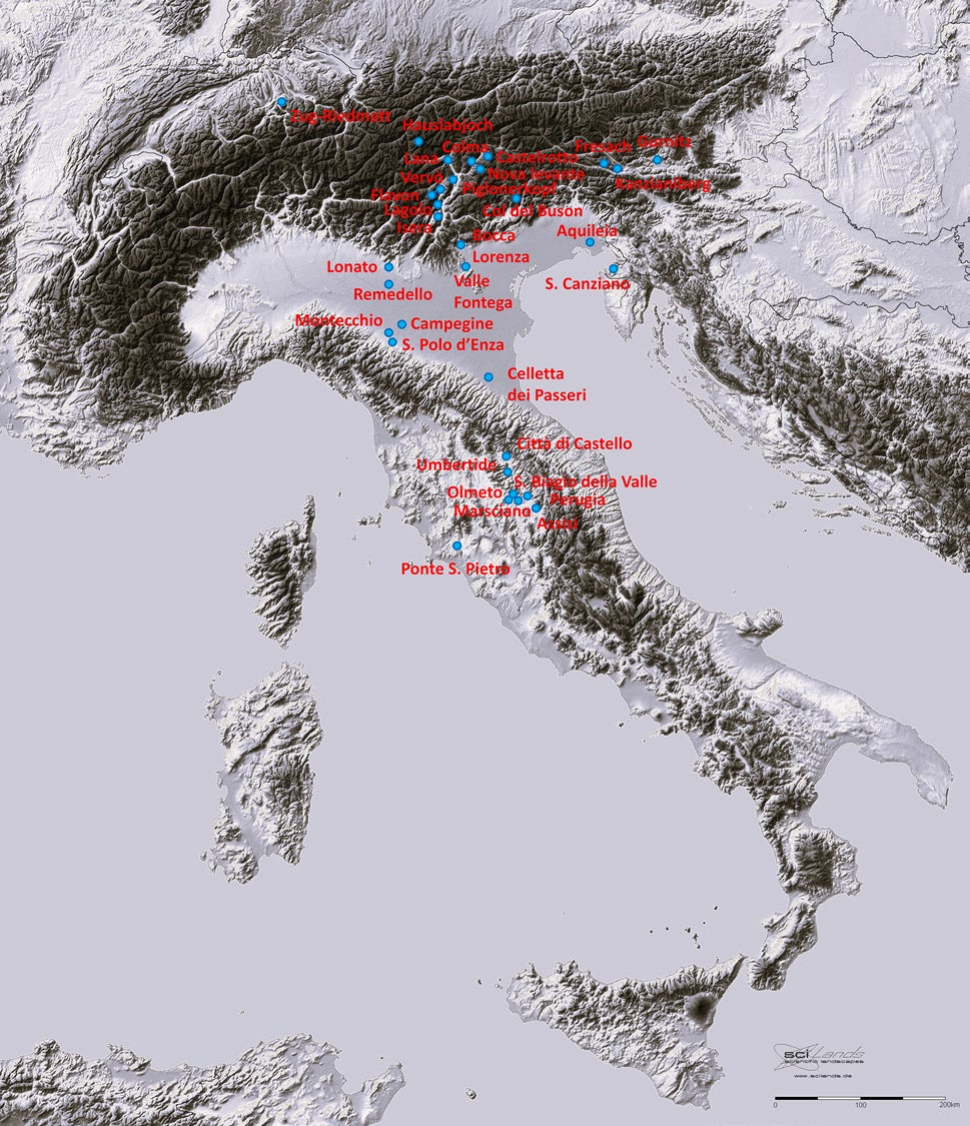
Geographical distribution of the copper objects investigated. (Relief map of Italy modified from Wikimedia Commons: upload.wikimedia.org/wikipedia/commons/4/40/449 × 562-GMT-Italie-R1.jpg free for reuse under the Wikimedia Commons terms).

The Alpine region is lacking world class copper deposits, such as the Michigan or Chilean ores, but it is pervaded by numerous small deposits of different origin, reflecting the complex geological process related to the Alpine-Himalayan Orogeny^18^. Many of the deposits were certainly of economic interest already in Roman times, though they started to be massively exploited in Late Medieval times^19^, when incoming German miners imported mining technology and metallurgical know-how from Saxony, starting from the Schwaz region in Austria and then spreading in Alto Adige/Südtirol (Pfunderer Berg), Trentino (Monte Calisio, Valle dei Mocheni) and Veneto (Valle Imperina). The widespread occurrence of Medieval mining and smelting activities, followed by heterogeneous and intermittent local exploitations through Renaissance till the Industrial revolution and modern times, concurred to create in local people a diffuse knowledge of long lasting mine heritage, which ended abruptly in the last decades of the twentieth century, due to globalization of the metal markets and economy. No metal mine is active in the whole area today. The generations-long memories of past metal activities also created the widespread narrative that the ore resources were also exploited in prehistoric and protohistoric times, although hard archaeological evidence of ancient mining and smelting activities are chronologically scattered and poorly supported by in depth analyses of circulating metal objects. For example, claims of 4th millennium mining in Libiola, Liguria^20^ and early 2nd millennium mining at Saint-Véran, Queiras^21^ have not yet been substantiated by analysis of coeval metal object carrying compatible patterns of chemical or isotopic tracers. On the other hand there is sound archaeological evidence of extensive smelting activities in the Alto Adige/Südtirol and Trentino areas during the Recent and Final Bronze Age^11,12,22^, essentially confined within the geographic boundaries defined by the Luco/Laugen Culture ceramics^23,24^, and this massive copper production in the Alps during the second half of the 2nd millennium BC was firmly confirmed by the large amount of coeval bronze objects carrying the Alpine lead isotopic signal^8,10^, scattered as far as Central Europe, Scandinavia and possibly Greece^25,26^. The same confirmation was attested for the Late Eneolithic smelting slags evidences found in archaeological excavations along the Isarco and Adige Rivers, and at the mouth of the Valsugana Valley^16,17^. They are all dated after the second quarter of the 3rd millennium BC, and the well-defined lead isotopic signal pertaining to South-Eastern Alpine ores was traced to several coeval copper artefacts (Fig. 6 and Table 4 of Ref.^27^). However, analyses of well-dated small awls and pins circulating during the end of the 5th millennium and the early part of the 4th millennium carry the isotopic signal of the Balkan deposits^8^, and those of several objects circulating during the last part of the 4th millennium BC, including the popular Iceman’s axe, indicated copper sources from Tuscany^14,15,28^. Therefore, in order to clarify which copper sources were active during the long Italian Copper Age (Eneolithic), and to pinpoint the time of the early adoption and use of Alpine copper, a specific project supported by the Museo Archeologico dell’Alto Adige/Südtiroler Archäologiemuseum was undertaken to analyse a substantial selection of important copper objects chronologically attributed to the Italian Copper Age, and present in the collection of several Museums in Italy and abroad. The detailed locations of finding, the Museum in which the objects are in storage/display, and the collection/catalogue number of the analysed objects are reported in the Supplementary Table 1. The geographical distribution of the objects analysed is reported in Fig. 1, together with a few other objects previously analysed^14,16,29^. All listed objects were characterised by lead isotope (LI) analysis, metallography by reflected light optical microscopy, and chemical analysis by electron probe microanalysis. The details of the sampling and measurement procedures are described in the Methods section, in Supplementary Materials. All the chemical and isotopic results are reported in the Supplementary Tables 2, 3 and 4.

### The chronological problem: dating vs seriation

The major issue confronted during the interpretation of the measured LI data lies in the challenge related to the chronological attribution of the objects. In fact, only a few of the objects have a secure chronological assignment due to security of context from careful stratigraphic excavation and radiocarbon dating of associated organic materials, usually the bones of the burial. The available radiocarbon dates are reported in the Supplementary Table 1. In the exceptional case of the Iceman’s axe direct radiocarbon dating of the implement was possible on the well-preserved wooden shaft^30,31^. Most of the other Eneolithic artefacts derive from century old excavations, carried out with no strict stratigraphic protocols, or they are stray finds with no secure context. For these objects only a general typological seriation is available and the data table reports the most accepted attribution^32–35^, although it should be noted that when absolute dating is lacking the chronological assignment are subject to debate. For the general assessment of sourcing the copper deposits, a simplified chronology dividing the entire Copper Age into three major periods is adopted, conventionally labelled Copper Age 1, Copper Age 2, and Copper Age 3, and finer subdivisions proposed by some scholars are here neglected. The attribution of absolute dates to these periods reported in Table 1 may be the subject of controversy^32–35^, but it is a fact that Copper Age 1 marks a neat cultural change from the earlier Neolithic period, as evidenced by some of the major Eneolithic Italian Cultures (the Rinaldone Culture in Central Italy^36^, the Spilamberto and Remedello Cultures in Northern Italy^37^, the Gaudo Culture in the Center-South). The start of the Copper Age 2 is historically linked to the changes observed between the Phase I and II in the necropolis of Remedello di Sotto, Brescia^37^, and the beginning of Copper Age 3 is roughly concomitant with the so-called Beaker event^38^.

**Table 1:** Interpretation of the copper axes based on Pb isotopic signature (provenance of copper) and assigned chronology from typological seriation.

Chronology	Balkan copper	Tuscanian copper	Alpine copper
Late Neolithic (4500–3500 BC)	Lana BZ (As, Zn, Ag) Nova Levante-Welschnofen BZ (As) Colma BZ (As) San Canziano SLO (Ag) Kanzianiberg AUT (Zn, Ag) Gurnitz AUT (As, Ag, Zn) Bocca Lorenza VI (Zn, Ag) (3 axes) San Polo d’Enza RE (As, Ag, Zn) Castelrotto-Kastelruth BZ (As) Campegine RE (pure Cu) Marsciano-Badiola PG (Ag, As) **Ponte San Pietro VT (As, Ag, Zn, Sb)**		Arcugnano-Valle Fontega VI (Zn, Ag)
Copper Age 1 (3500–3000 BC)	Montecchio Emilia-La Sacca RE (Cu)	Lonato BS (Ni) Remedello Tb 4 BS (As) Remedello Tb 102 BS (As, Ag, Zn) Perugia/Pila PG (As, Sb) Perugia PG (As, Sb) Perugia PG (Territorio) (Zn, Ag) Olmeto PG (Zn, As, Sb) Assisi San Martino PG (Zn, As) Umbertide PG (As, Zn) Città di Castello PG (As, Ag, Sb) S. Biagio della Valle PG (Zn, As, Ag, Sb) **Tisenjoch-Similaun BZ** **Zug-Riedmatt CH**	Vervò TN (Ag, As, Sb) Aquileia TS (Zn, Ag)
Copper Age 2 (3000–2500 BC)		Remedello Tb 62 BS (As, Ag, Zn) **Remedello Tb 78 BS (As, Ag, Zn)** Celletta dei Passeri Tb 40 FC (As, Ag, Zn) **Celletta dei Passeri Tb 47 FC (As, Ag, Zn)**	Flavon TN (Sb) Lagolo TN (Cu)
Copper Age 3 (2500–2200 BC)			Col del Buson BL (Cu) **Pigloner Kopf BZ (Zn, Pb) (4 axes)** Fresach-Villach AUT (Zn, As)

The axes from the few localities highlighted in bold were directly or indirectly radiocarbon dated (see Supplementary Table 1). The axes are identified by the find localities as marked in Fig. 1, together with the administrative province and the measured chemical impurities in the metal (in brackets).

### The copper ore sources: measured data

The interpretation of the copper source is essentially based on the comparison of the measured lead isotope (LI) ratios with those of copper ores compiled in the existing databases^7–9,27^. The LI provenance is then confronted with elemental impurities measured in the copper metal in order to confirm that the mineral assemblage of the potential ore source is compatible with the observed chemistry of the sample.

All objects investigated in this study were chemically analysed by scanning electron microscopy (SEM) equipped with energy dispersive spectrometry (EDS), and by electron probe microanalysis (EPMA) that provides lower limits of detection for most detected elements (see Supplementary Materials, Tables 2 and 3). The lead isotope ratios of all samples (Supplementary Materials, Table 4) were measured by multi-collector plasma source mass spectrometry (MC-ICP-MS).

**Figure 2:**
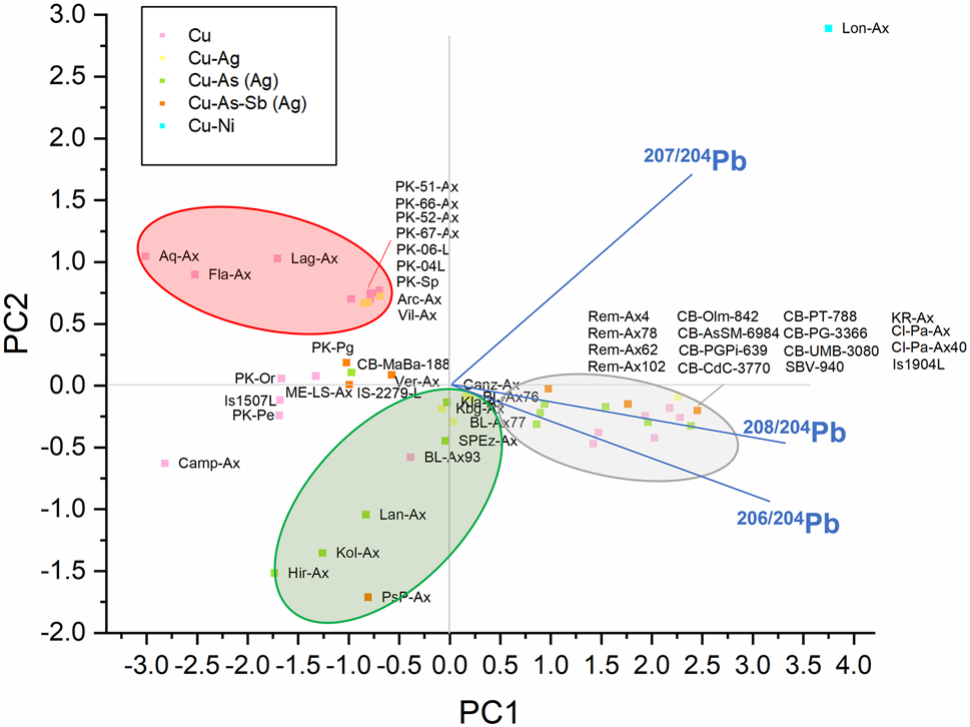
Principal component analysis (PCA) of the isotopic data measured on the Late Neolithic and Eneolithic copper artefacts, both axes and other objects. Each object is marked with the sample label reported in the Supplementary Table 1.

**Figure 3:**
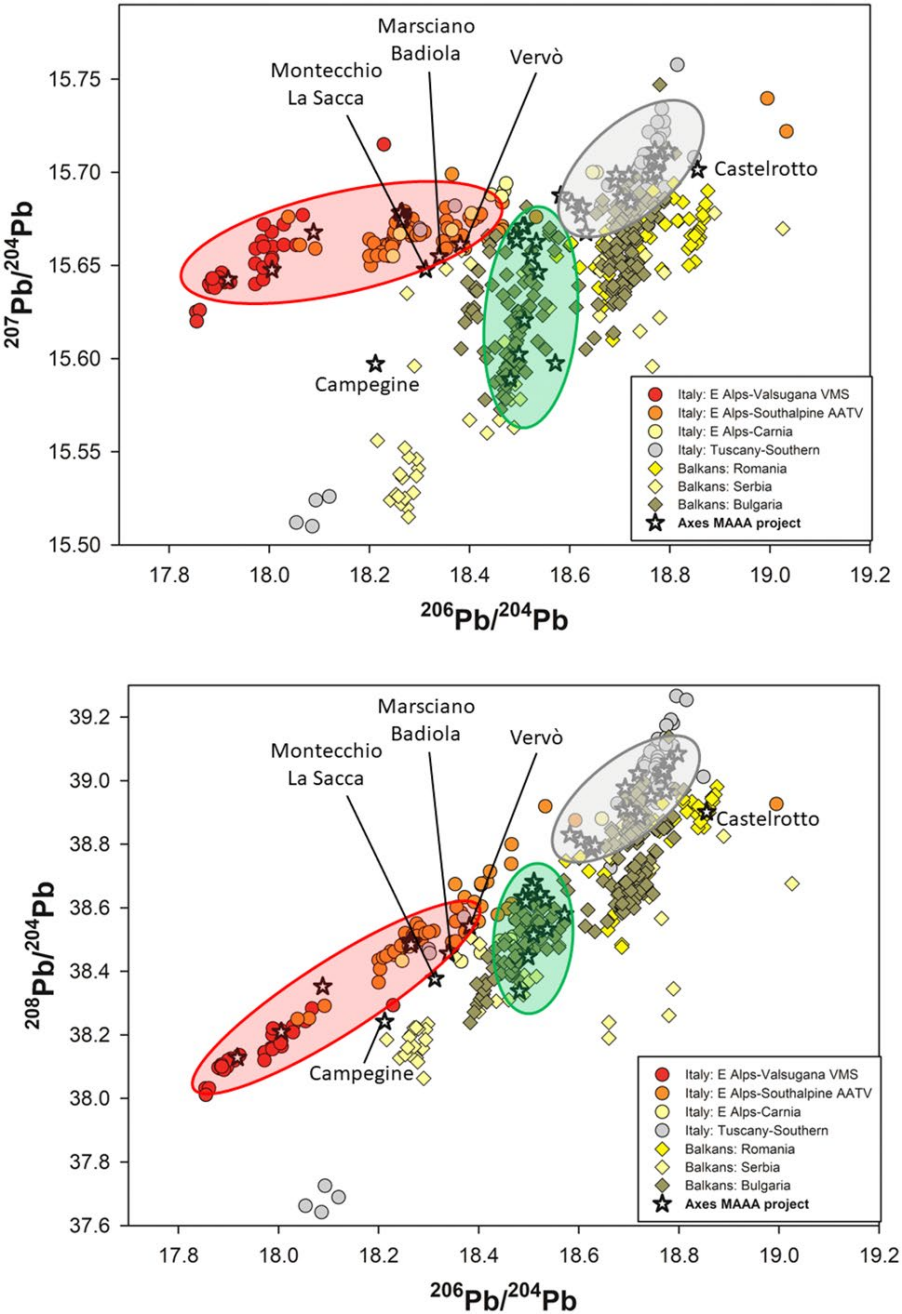
2D projections of the 3D isotopic space, showing the correspondence between the ore fields in the database and the measured LI ratios in the copper axes. The names of the ore fields follow the ones proposed in the literature^27^.

The LI measured on the samples selected for the project (see Supplementary Table 4) were also analysed by principal component analysis (PCA), in order to widen our exploratory data analysis. Note that, even if the LIA data do not strictly follow the mathematical "constant sum requirement"^39^, the PCA can provide additional support to the primary LIA data^40^. Figure 2 shows that, as expected, the samples cluster into three major groups corresponding closely to three of the most copper-rich regions exploited in the past: (a) the Alpine region (red ellipse), marked by low values of ^206^Pb/^204^Pb and ^208^Pb/^204^Pb, (b) Tuscany (grey ellipse), marked by high values of ^206^Pb/^204^Pb and ^208^Pb/^204^Pb, and (c) the Balkans (green ellipse), marked by low values of ^207^Pb/^204^Pb. The general good correspondence between the PCA clusters of the objects and the ore fields is directly visible in the 2D projections of the database isotopic data of the ores (Fig. 3). The few objects that are plotting outside the ellipse areas of the ore fields both in the PCA and LI data plots are mostly not axes, and the few axes deviating from the main fields are discussed individually.

### The copper ore sources: data discussion

If all the measured copper axes are listed together with the attributed chronology (Table 1), a very straightforward and interesting pattern is observed: in each chronological period there is a very distinctive use of a specific ore source. The few objects at variance with the model will be discussed in detail. Virtually, all Late Neolithic axes were manufactured and imported from the Balkans, where copper resources were exploited starting from the 5th millennium BC^41^. It is very interesting to note that based on the axes circulating in Italy, several copper sources were already active in the Balkans. Most of the axes were made out of Bulgarian ores (green ellipse in Figs. 2, 3), in good agreement with the signal of the circulating axes in the Balkans (see Supplementary Fig. 1), but one axe has affinity with Serbian ores (Campegine, despite the large estimated uncertainty of the measured LIA data due to the very low amount of Pb contained in the copper), and another one to Rumanian ores (Castelrotto-Kastelruth). The axes from Marsciano-Badiola and Montecchio-La Sacca are at the boundary of the Alpine ore fields, and they are definitely out of the isochron line defining the Alpine model age. For these two axes the affinity to the Serbian ores is also supported by the early typology of the objects. These axes interpreted as made of Serbian copper lay in between the Alpine and Balkan fields in the PCA diagram of Fig. 2, together with two of the Late Neolithic awls from Isera (samples Is-1507-L and Is-2279-L). It should be noted that the pattern here observed for the Late Neolithic axes matches closely the one observed for the coeval awls of the late 5th millennium–early 4th millennium BC (Fig. 1 of Ref.^8^, now revised as Supplementary Fig. 2 with the data published after 2020), which are all composed of Balkanic copper. The axe from Arcugnano-Valle Fontega apparently indicate a very early occurrence of Alpine copper, though it is here excluded from the discussion, because it is an incomplete axe, evidently recomposed from several fragments of different origin and therefore typologically very dubious. It cannot be safely assigned chronologically.

Following this early period of import of Balkanic copper objects, the Italian cultures of the second half of the 4th millennium BC (Copper Age 1: Rinaldone, Remedello) are systematically making use of Tuscanian copper, which proved to travel as far North as Switzerland (Zug-Riedmatt axe^28^) and South Tyrol (Tisenjoch-Similaun axe^14^). Interestingly, the oldest object in the Ponte San Pietro necropolis, the notorious axe of the “Tomba della Vedova” is made of Balkanic copper, whereas all the other axes of the period are made of local Tuscanian copper. The axe from tomb n.20 of Ponte San Pietro belongs to the male, dated 3540–3360 cal. BC^42^. Therefore, copper from Balkans area was still circulating when Tuscan copper was already exploited. The primary production of copper in Southern Tuscany in the Copper Age 1 period is well attested by the metallurgical site of S. Carlo^43^. The Tuscanian copper seems to dominate the production and trade of copper axes, although objects made of non-Tuscanian copper have been reported from the area^15^, possibly made in the region as attested by the typology of the objects. It is interesting that the Tuscanian copper is ubiquitously present even in the later phases of the Remedello-related necropolises (Remedello di Sotto^37^, Celletta dei Passeri^44^) that are securely referred to the first centuries of the 3rd millennium BC (see Supplementary Table 1). The only object typologically attributed to Copper Age 1 and apparently showing an isotopic signal compatible with the Alpine ores is the axe from Vervò (Fig. 3). Should this interpretation be correct, it would signal a rather early start of exploitation of Alpine ore deposits, of which there is no direct evidence in terms of mines and metallurgical activities, such as ore beneficiation or smelting. An alternative interpretation of the Pb isotope signal of the Vervò axe may be proposed, which fits with the model of copper production in Tuscany in the second half of the 4th millennium BC: the copper of the Vervò axe could have been extracted from the ores of the Apuanian Alps. In fact, the isotopic character of the Apuanian Alps ores is fully compatible with that measured on the axe (see Supplementary Fig. 3), although this would represent the earliest evidence of exploitation of Northern Tuscanian ores. This interpretation would also account for the substantial amount of Sb present in the copper of the Vervò axe (Supplementary Tables 2 and 3), which is compatible with the mineralogy of the Apuanian Alps ores, but incompatible with the South Eastern Alpine deposits. 

The LI evidence therefore indicates a massive copper production in Tuscany during the whole second half of the 4th millennium (Copper Age 1), and the first part of the 3rd millennium (Copper Age 2). The model points to persisting relations between the populations inhabiting the Po River valley and those of Central Italy, at a time when the Alpine ores start to be mined and exploited for the manufacturing of tools, as indicated by several axes analysed in the present project and attributed to Copper Age 2 (Table 1: Flavon, Lagolo, possibly Aquileia) and by several awls of the 3rd millennium BC reported in the literature (Fig. 2 of Ref.^8^).

Starting from the second half of the 3rd millennium (Copper Age 3) several Alpine areas are clearly producing copper, both from the As-containing copper ores of the Valsugana Valley, and from other chalcopyrite-based deposits of the Trentino-Alto Adige (Fig. 3). The coeval axes of Col del Buson in the Piave Valley are consistent with this interpretation^29^. The Alpine copper production seems to dominate the copper market in this period, as shown by the exclusive presence of Alpine copper in all analysed objects of the period (Table 1), and by the diffuse presence of copper smelting slags in the South-Eastern Alps (Isarco River valley, Adige valley)^16,17^. This massive production is chronologically synchronous to the so-called Beaker event, which is related to the increase of metallurgical activities in other parts of the European continent^45,46^.

### The flow of copper in Late Neolithic and Eneolithic Italy

**Figure 4:**
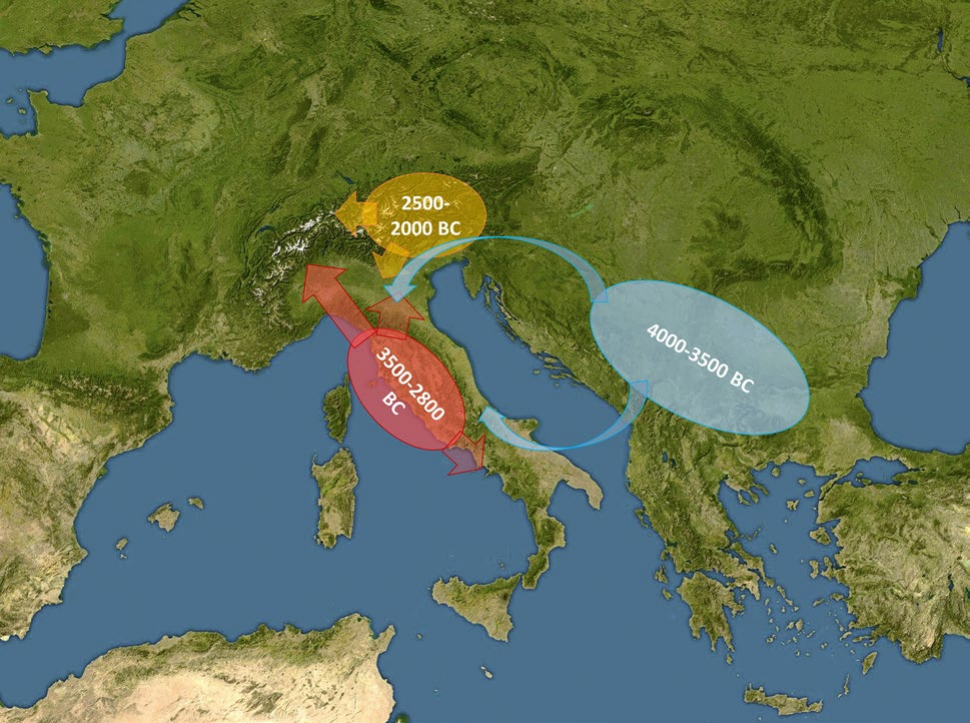
Diagram showing the diffusion of copper in Northern and Central Italy during the 4th and 3rd millenniums BC. (Map modified from the Pixabay repository: pixabay.com/illustrations/europe-map-satellite-image-1290868/ free for use and download under the Pixabay terms of service).

The measurement of the LI signal in selected prehistoric copper objects, mostly axes, spanning from the mid-5th to the 3rd millennium BC allowed to identify the ore sources and the flow of the metal across Northern and Central Italy. Although some of the chronological attributions to the analysed objects may be discussed, the picture derived from the analyses shows clearly that distinct ore deposits were exploited during the archaeologically-defined periods since the late Neolithic, with minimal overlap during transitional periods (Fig. 4).

The earliest metals were invariably small objects made with Balkanic copper, extracted in Serbia, Bulgaria and, possibly Romania. Tuscanian copper starts to flow around the mid of the 4th millennium BC and rapidly diffuses in the whole Central and Northern Italy, and beyond. The total lack of ingots from this period hints that the artefacts were transported and traded, not the raw metal. During the Copper Ages 1 and 2 (i.e. till the first part of the 3rd millennium) Tuscanian copper dominates the use of metal, and there is no firm evidence of exploitation of the Alpine deposits, which actually started to yield copper only around the middle of the 3rd millennium BC, in connection or slightly earlier than the Beaker cultural event. The Alpine LI signal found in the objects confirms the abundant archaeological evidence of smelting activities excavated along the Isarco and Adige Rivers Valleys^16^, dated to Copper Age 3.

The relative role of the Tuscanian and Alpine productions during the Copper Age-Bronze Age transition is unclear. Certainly, the Early Bronze Age populations of the Po River Valley took advantage of the copper inflow both from the North and the South, although the factors controlling the quantity of copper production in the two areas are still debated. The arrival of tin in the Alpine region around the turn of the millennium could be the key factor shifting the massive production of metal to the South-Eastern Alps, which became the most predominant copper source in the Bronze Age.

### Supplementary Information

Supplementary Information.

## Data Availability

All data generated or analysed during this study are included in this published article and its supplementary information files.
