# Supplementary material for: Lead isotopes of prehistoric copper tools define metallurgical phases in Late Neolithic and Eneolithic Italy
Source: Sci Rep. 2024 Feb 21;14:4323. doi: 10.1038/s41598-024-54825-z (PMC10881475; doi:10.1038/s41598-024-54825-z)
Supplement: Supplementary file 1 — Supplementary Information. [file 41598_2024_54825_MOESM1_ESM.pdf]

Supplementary Information

Lead isotopes of prehistoric copper tools define metallurgical phases in Late Neolithic and Eneolithic Italy

Gilberto Artioli, Ivana Angelini, Caterina Canovaro, Guenther Kaufmann, Igor Maria Villa

Methods

After a careful observation under a stereomicroscope, a few micro-fragments (4-5 mg) were detached from the objects using a fine steel blade, paying attention to extract fresh metal, without oxidized portions and avoiding surface contaminations. One of the micro–samples was embedded in epoxy resin, preserving the original orientation in the object, and used to perform chemical analyses on the metal matrix, inclusions and segregations by SEM-EDS and in order to fully characterized the material of each artifacts.
For the provenance study by Lead Isotopic Analysis (LIA), the second aliquot of metal detached from the objects was pre-treated and dissolved in the ultraclean room installed at the Department of Geosciences, University of Padova. The samples were dissolved in hot triply distilled concentrated nitric acid in screw-top PTFE (polytetrafluoroethane) vessels. As described in ref.[1], the dissolved lead was purified using the SrSpec™ resin (EIChroM Industries). Lead isotope ratios were measured by multi-collector plasma source mass spectrometry (MC-ICP-MS) using a Thermo Scientific NeptunePlus instrument at the Institut für Geologie, University of Bern (Switzerland). The Faraday collector array allows the simultaneous acquisition of masses 202 to 209. The sample introduction system consisted of an auto-aspirating low-flow (50 µl min–1) Apex desolvating nebulizer (ESI Scientific, Omaha, NE, USA) mounted on to a combined cyclonic/double-pass spray chamber made of quartz glass. Potential isobaric interference of ^{204}Hg on ^{204}Pb was controlled and, if necessary, corrected for by monitoring the ^{202}Hg signal. Hydride formation (PbH+) was monitored on mass 209 and never detected. Mass fractionation was monitored by adding a small quantity of Tl, which has a known ^{203}Tl/^{205}Tl ratio, is fractionated by the same mechanism as Pb and does not interfere with Pb isotope measurements[2]. Typical in-run relative uncertainties (2 SE of the mean) on ^{206}Pb/^{204}Pb, ^{207}Pb/^{204}Pb, and ^{208}Pb/^{204}Pb isotope ratios ranged between 0.004 and 0.02 %. The measurement accuracy was controlled with frequent measurements of the NIST SRM 981 standard reference material interspersed with the sample measurements. The measured isotopic composition for SRM 981 were indistinguishable from the certified value and the recent more precise literature measurements[3], so that no adjustment of the measured ratios was necessary. The external reproducibility on the SRM 981 reference material over the measuring period of several months amounted to ± 100 ppm, or 0.01 % (2σ), very similar to the individual in-run precision on unknown samples.

Supplementary Table 1

List of copper samples analysed, with relevant reference information. Information about the find context, category of items, typology and chronology of all the analyzed objects within this study. Column 7 is from the most recent typological revisions, though the chronological assignment may be subject to debate. Column 8 reports the available calibrated 14C dates at the 2σ level (95.4 % confidence).

	Sample label	Location of finding	Location of the object	Catalogue n.	Description	Chronology assigned from typology	Chronology from ^{14}C dating
1	Aq-Ax	Aquileia (UD)	Museo Archeologico Nazionale di Aquileia	AQ 23209	Flanged axe	Copper Age 1-2	
2	Arc-Ax	Arcugnano, Valle Fontega (VI)	Museo Naturalistico Archeologico, Vicenza	n. 613 IG 163527	Flat axe	Recent Neolithic	
3	BL-Ax76	Santorso/Bocca Lorenza (VI)	Museo Naturalistico Archeologico, Vicenza	n. 7776	Flat axe	Recent-Late Neolithic	
4	BL-Ax77	Santorso/Bocca Lorenza (VI)	Museo Naturalistico Archeologico, Vicenza	n. 7777	Flat axe	Recent-Late Neolithic	
5	BL-Ax93	Santorso/Bocca Lorenza (VI)	Museo Naturalistico Archeologico, Vicenza	n. 4393 IG 162415	Flat axe	Recent-Late Neolithic	
6	Camp-Ax	Campegine (RE)	Museo Civico di Modena	n. 1519	Flat axe	Recent Neolithic	
7	Canz-Ax	San Canziano, Grotta Tominz (Slovenia)	Museo d'antichità J.J. Winckelmann, Trieste	GR 0519	Flat axe	Recent-Late Neolithic	
8	CB-AsSM-6984	Assisi/San Martino (PG)	Museo Archeologico Nazionale dell’Umbria, Perugia	n. 721660 (coll. Bellucci 6984)	Flat axe	Recent Neolithic	
9	CB-CdC-3770	Città di Castello (PG)	Museo Archeologico Nazionale dell’Umbria,	n. 721684 (coll. Bellucci 3770B)	Flanged axe	Copper Age 1-2	
10	CB-MaBa-188	Marsciano, Badiola (PG)	Museo Archeologico Nazionale dell’Umbria, Perugia	n. 721654 (coll Bellucci 188)	Flat axe	Recent Neolithic	
11	CB-OLM-842	Olmeto (PG)	Museo Archeologico Nazionale dell’Umbria,	n. 721681 (coll. Bellucci 842B)	Flanged axe	Copper Age 1-2	
12	CB-PG-3366	Perugia (PG)	Museo Archeologico Nazionale dell’Umbria,	n. 721656 (coll Bellucci 3366)	Flat axe	Recent Neolithic	
13	CB-PGPi-639	Perugia, Pila (PG)	Museo Archeologico Nazionale dell’Umbria, Perugia	n. 721655 (coll Bellucci 639)	Flat axe	Recent Neolithic	
14	CB-PT-778	Perugia (PG)	Museo Archeologico Nazionale dell’Umbria, Perugia	n. 721665 (coll. Bellucci 778B)	Flanged axe	Copper Age 1-2	
15	CB-UMB-3080	Umbertide (PG)	Museo Archeologico Nazionale dell’Umbria,	n. 721683 (coll. Belucci 3080B)	Flanged axe	Copper Age 1-2	
16	Cl-Pa-Ax	Forlì, Celletta dei Passeri, (FC)	Soprintendenza di Forlì-Cesena, deposito di Faenza	Tb. 47 n. 261204	Flanged axe	Copper Age 2	3010-2660 BC[7]

17	Cl-Pa-Ax40	Forlì, Celletta dei Passeri, (FC)	Soprintendenza di Forlì-Cesena, deposito di Faenza	Tb. 40 n.261194	Flanged axe	Copper Age 2	
18	Fla-Ax	Contà, Flavon (TN)	Castello del Buonconsiglio, Trento	n. 3516	Flanged axe	Copper Age 2	
19	Hir-Ax	Nova Levante-Welschnofen, Hirzlsteig (BZ)	Ufficio Beni Archeologici, Bolzano	SAM Kz3	Flat axe	Late Neolithic – Copper Age 1	
20	Is-1507-L	Isera, La Torretta (TN)	Soprintendenza per i beni e le attività culturali della Provincia autonoma di Trento - Ufficio Beni archeologici	RR 1507	Awl, rectangula r section	Late Neolithic	Post 4367-4218 BC[4]
21	Is-1904-L	Isera, La Torretta (TN)	Soprintendenza per i beni e le attività culturali della Provincia autonoma di Trento - Ufficio Beni archeologici	RR 1904	Awl, square section	Late Neolithic	Post 4367-4218 BC[4]
22	Is-2279-L	Isera, La Torretta (TN)	Soprintendenza per i beni e le attività culturali della Provincia autonoma di Trento - Ufficio Beni archeologici	RR 2279	Folded copper sheet	Recent Neolithic	Post 4367-4218 BC[4]
23	KBG-Ax	Finkenstein am Faaker See, Kanzianiberg (Austria)	Museum der Stadt, Villach	AR 105	Flat axe	Recent-Late Neolithic	
24	Kla-Ax	Ebemtal, Gurnitz (Austria)	Landesmuseum Kärnten, Klagenfurt	n. 2377	Flat axe	Recent-Late Neolithic	
25	Kol-Ax	Barbian-Barbiano, Kollmann-Colma (BZ)	Museo Archeologico dell’Alto Adige, Bolzano	SAM Kz1	Flat axe	Late Neolithic – Copper Age 1	
26	Kr-Ax	Castelrotto-Kastelruth, Gamertinerhof (BZ)	Ufficio Beni Archeologici, Bolzano	SAM Kz2	Flat axe	Late Neolithic – Copper Age 1	
27	Lag-Ax	Lasino, Lagolo (TN)	Fondazione Museo Civico di Rovereto	n. 3187 (ex. 750)	Flanged axe	Copper Age 2	
28	Lan-Ax	Lana, Gaulschlucht (BZ)	Museo Mamming, Merano	n. 92	Flat axe	Recent-Late Neolithic	
29	Lon-Ax	Lonato del Garda (BS)	Museo delle Civiltà Pigorini, Roma	n. 60038	Flanged axe	Copper Age 1	
30	ME-LS-Ax	Montecchio Emilia, La Sacca (RE)	Museo Gaetano Chierici di Paletnologia, Reggio Emilia	n. 25-8	Flanged axe	Copper Age 1	
31	PK-04-L	Vadena-Pfatten, Pigloner Kopf (BZ)	Museo Archeologico dell’Alto Adige, Bolzano	Rz 11504	Awl	Copper Age 3	2464-2298 BC[8]
32	PK-06-L	Vadena-Pfatten, Pigloner Kopf (BZ)	Ufficio Beni Archeologici, Bolzano	Rz 1806	Awl	Copper Age 3	2464-2298 BC[8]

33	PK-51-Ax	Vadena-Pfatten, Pigloner Kopf (BZ)	Ufficio Beni Archeologici, Bolzano	n. 19152 – Axt2	Eye axe	Copper Age 3	2464-2298 BC[8]
34	PK-52-Ax	Vadena-Pfatten, Pigloner Kopf (BZ)	Ufficio Beni Archeologici, Bolzano	n. 19152 – Axt3	Eye axe	Copper Age 3	2464-2298 BC[8]
35	PK-66-Ax	Vadena-Pfatten, Pigloner Kopf (BZ)	Ufficio Beni Archeologici, Bolzano	Rz 1666	Eye axe	Copper Age 3	2464-2298 BC[8]
36	PK-67-Ax	Vadena-Pfatten, Pigloner Kopf (BZ)	Ufficio Beni Archeologici, Bolzano	Rz 1667	Eye axe	Copper Age 3	2464-2298 BC[8]
37	PK-Or	Vadena-Pfatten, Pigloner Kopf (BZ)	Museo Archeologico dell’Alto Adige, Bolzano	Rz 11466	Coiled spiral	Copper Age 3	2464-2298 BC[8]
38	PK-Pe	Vadena-Pfatten, Pigloner Kopf (BZ)	Museo Archeologico dell’Alto Adige, Bolzano	Rz 11467	Double spiralled pendant	Copper Age 3	2464-2298 BC[8]
39	PK-Pg	Vadena-Pfatten, Pigloner Kopf (BZ)	Museo Archeologico dell’Alto Adige, Bolzano	Rz 19158	Dagger	Copper Age 3	2464-2298 BC[8]
40	PK-Sp	Vadena-Pfatten, Pigloner Kopf (BZ)	Museo Archeologico dell’Alto Adige, Bolzano	Rz 11465	Coiled ornament	Copper Age 3	2464-2298 BC[8]
41	PsP-Ax	Ponte San Pietro (VT)	Museo delle Civiltà Pigorini, Roma	Tb. 20 “della vedova” n. 111786	Flanged axe	Copper Age 1	3540-3360 BC[5]
42	Rem-Ax102	Remedello di Sotto, Dovarese (BS)	Museo Gaetano Chierici di Paletnologia, Reggio Emilia	Tb. 102 n. 24798	Flanged axe	Copper Age 1	
43	Rem-Ax4	Remedello di Sotto, Dovarese, (BS)	Museo Gaetano Chierici di Paletnologia, Reggio Emilia	Tb. 4	Flanged axe	Copper Age 2	
44	Rem-Ax62	Remedello di Sotto, Dovarese, (BS)	Museo Gaetano Chierici di Paletnologia, Reggio Emilia	Tb. 62	Flanged axe	Copper Age 2	
45	Rem-Ax78	Remedello di Sotto, Dovarese, (BS)	Museo Gaetano Chierici di Paletnologia, Reggio Emilia	Tb. 78 n. 24675	Flanged axe	Copper Age 2	3090-2900 BC[6]
46	SBV-940	Casanuova di san Biagio della Valle (PG)	Museo Archeologico Nazionale dell’Umbria,	n. 1400940/3	Flanged axe	Copper Age 1-2	
47	SPEz-Ax	San Polo d'Enza (RE)	Museo Gaetano Chierici di Paletnologia, Reggio Emilia	n. 25-9	Flanged axe	Copper Age 2	
48	Ver-Ax	Vervò (TN)	Ufficio Beni Archeologici, Trento	n.a.	Flanged axe	Copper Age 1-2	
49	Vil-Ax	Fresach (Austria)	Museum der Stadt, Villach	AR 193	Eye axe	Copper Age 3	

Supplementary Table 2

Mean chemical composition of the analysed copper objects, measured by area mode SEM-EDS. Each result is an average of three measurements (sd= standard deviation). Values are in wt % of the element. All analyses renormalized to 100.

Sample	O K	CuK	FeK	S K	PbM	NiK	AsK	SbL	AgL	BiL	ClK
**Aq-Ax**		99.8			0.2						
sd		*0.2*			*0.2*						
**Arc-Ax**		98.9			1.1						
sd		*0.2*			*0.2*						
**BL-Ax76**		97.4	0.1		0.1	0.3			2.1		
sd											
**BL-Ax-77**	0.6	99.4									
sd	*0.1*	*0.1*									
**BL-Ax-93**	0.6	99.4									
sd	*0.1*	*0.1*									
**Camp-Ax**	0.5	99.5									
sd	*0.1*	*0.1*									
**Canz-Ax**		98.5							1.5		
sd		*0.2*							*0.2*		
**CB-AsSM-6984**		98.8			1.2						
sd		*0.1*			*0.1*						
**CB-CdC-3770**	1	99									
sd	*0.1*	*0.1*									
**CB-MaBa-188**		99.1							0.9		
sd		*0.2*							*0.2*		
**CB-OLM-842**		99						1			
sd		*0.1*						*0.1*			
**CB-PG-3366**	1.1	98.9									
sd	*0.1*	*0.1*									
**CB-PGPi-639**	1.1	98.9									
sd	0.1	0.1									
**CB-PT-778**	1.4	98.6									
sd	*0.1*	*0.1*									
**CB-UMB-3080**		97.8					2.2				
sd		*0.2*					*0.2*				
**Cl-Pa-Ax**		99.7					0.3				
sd		*0.3*					*0.3*				
**Cl-Pa-Ax40**	0.9	99.1									
sd	*0.1*	*0.1*									
**Fla-Ax**		99.3						0.7			
sd		*0.1*						*0.1*			
**Hir-Ax**		98.4					1.6				
sd		*0.2*					*0.2*				
**Is-1507-L**	0.9	99.1									
sd	*0.1*	*0.1*									
**Is-1904-L**		99.3	0.7								
sd		*0.3*	*0.3*								
**Is-2279-L**		97.6			1.1			1.3			
sd		*0.9*			*0.3*			*0.6*			
**KBG-Ax**	0.9	99.1									
sd	*0.1*	*0.1*									
**Kla-Ax**		96.9					1.7		1.4		
sd		*0.5*					*0.1*		*0.6*		

**Kol-Ax**<br>*sd*		96.3<br>*0.4*					3.7<br>*0.4*		0.1<br>*0.1*		
**Kr-Ax**<br>*sd*	0.8<br>*0*	98.7<br>*0.1*			0.5<br>*0.1*						
**Lag-Ax**<br>*sd*	1.2<br>*0.1*	98.8<br>*0.1*									
**Lan-Ax**<br>*sd*	0.8<br>*0*	99.2<br>*0*									
**Lon-Ax**<br>*sd*		98.8<br>*0.2*				1.2<br>*0.2*					
**ME-LS-Ax**<br>*sd*	0.8<br>*0*	99.2<br>*0*									
**PK-04-L**<br>*sd*		99.3<br>*0.4*			0.7<br>*0.4*						
**PK-06L**<br>*sd*		98.3<br>*0.3*			0.6<br>*0.6*						1.1<br>*0.3*
**PK-51-Ax**<br>*sd*		98.6<br>*0.2*		0.4<br>*0.1*	1<br>*0.1*						
**PK-52-Ax**<br>*sd*		98.5<br>*0.2*		0.4<br>*0.2*	1.1<br>*0.1*						
**PK-66-Ax**<br>*sd*		98.4<br>*0.1*		0.6<br>*0.1*	1<br>*0.2*						
**PK-67-Ax**<br>*sd*		98.4<br>*0.6*		0.2<br>*0.1*	1.4<br>*0.5*						
**PK-Or**<br>*sd*		98.7<br>*0.3*			1.3<br>*0.3*						
**PK-Pe**<br>*sd*		99.2<br>*0.1*			0.8<br>*0.1*						
**PK-Pg**<br>*sd*		97.8<br>*0.5*			1.2<br>*0.3*			0.9<br>*0.2*			
**PK-Sp**<br>*sd*		98.9<br>*0.1*			1.1<br>*0.1*						
**PsP-Ax**<br>*sd*	1.2<br>*0.1*	98.8<br>*0.1*									
**Rem-Ax102**<br>*sd*		99.2<br>*0.1*					0.8<br>*0.1*				
**Rem-Ax4**<br>*sd*		98.8<br>*0.3*					1.2<br>*0.3*				
**Rem-Ax62**<br>*sd*		99.1<br>*0.2*					0.9<br>*0.2*				
**Rem-Ax78**<br>*sd*	1<br>*0.1*	99<br>*0.1*									
**SBV-940**<br>*sd*		97.6<br>*0.2*					1.5<br>*0.2*	0.9<br>*0*			
**SPEz-Ax**<br>*sd*		98<br>*0.1*					2<br>*0.1*				
**Ver-Ax**<br>*sd*		97.4<br>*1.2*					0.7<br>*0.3*	0.7<br>*0.4*	1.2<br>*0.6*		
**Vil-Ax**<br>*sd*		98.4<br>*0.2*			1.6<br>*0.2*						

Supplementary Table 3

Mean chemical composition of the analysed copper objects, measured by EPMA. Each result is an average of three measurements (sd= standard deviation). Values are in wt % of the element (n.a.= not analysed; b.d.l.=below the detection limit).

Label	S	Cl	Mn	Fe	Co	Ni	Cu	Zn	As	Ag	Sn	Sb	Pb	Bi	Tot
**Aq-Ax**	0.02	b.d.l.	0.01	0.01	0.01	0.01	99.84	0.09	0.03	0.08	0.01	0.07	0.03	0.02	100.23
sd	0.01		0.02	0.01	0.01	0.02	0.33	0.08	0.04	0.03	0.02	0.05	0.04	0.02	
**Arc-Ax**	0.02	b.d.l.	b.d.l.	0.01	0.02	0.02	100.2	0.11	0.06	0.23	0.02	0.03	0.05	0.01	100.73
sd	0.02			0.01	0.03	0.02	0.71	0.08	0.06	0.07	0.03	0.04	0.05	0.02	
**BL-Ax76**	0.01	0.01	b.d.l.	0.03	b.d.l.	0.01	99.53	n.a.	b.d.l.	0.85	b.d.l.	b.d.l.	0.04	b.d.l.	100.48
sd	0.01	0.01		0.02		0.01	0.16			0.13			0.04		
**BL-Ax77**	0.05	b.d.l.	0.01	0.01	0.01	0.02	99.32	0.12	0.04	0.62	0.01	0.01	0.01	0.04	100.29
sd	0.03		0.01	0.02	0.01	0.03	0.47	0.05	0.03	0.13	0.02	0.02	0.02	0.05	
**BL-Ax93**	0.01	b.d.l.	0.01	b.d.l.	b.d.l.	0.01	100.2	0.11	0.02	0.05	0.01	b.d.l.	0.04	0.03	100.54
sd	0.01		0.01			0.01	0.39	0.10	0.03	0.05	0.02		0.06	0.03	
**Camp-Ax**	0.02	0.01	0.01	0.01	b.d.l.	0.02	100.2	0.03	0.01	0.03	0.03	b.d.l.	0.03	0.01	100.38
sd	0.01	0.01	0.01	0.01		0.03	0.18	0.03	0.01	0.05	0.04		0.03	0.03	
**Canz-Ax**	0.02	0.01	0.01	b.d.l.	0.01	0.04	99.52	0.02	0.02	0.91	0.01	0.02	b.d.l.	b.d.l.	100.58
sd	0.02	0.01	0.01		0.01	0.03	0.19	0.04	0.03	0.12	0.01	0.04			
**CB-AsSM-6884**	0.01	b.d.l.	0.01	0.01	0.02	0.02	100.2	0.08	0.08	0.01	0.02	0.03	b.d.l.	0.03	100.5
sd	0.01		0.02	0.01	0.01	0.02	0.27	0.06	0.06	0.01	0.03	0.03		0.04	
**CB-CdC-3770**	0.01	b.d.l.	0.01	0.01	0.01	0.01	99.76	0.04	0.1	0.04	0.05	0.08	0.01	0.07	100.22
sd	0.01		0.01	0.02	0.02	0.02	0.14	0.04	0.05	0.03	0.02	0.06	0.02	0.07	
**CB-MaBa-188**	0.01	b.d.l.	0.02	0.01	0.01	0.01	98.75	0.05	0.65	0.86	0.01	0.02	0.03	0.04	100.47
sd	0.01		0.02	0.01	0.01	0.02	0.41	0.05	0.2	0.34	0.03	0.02	0.04	0.01	
**CB-OLM-842**	0.03	0.01	b.d.l.	0.01	0.01	0.05	99.79	0.09	0.26	0.03	0.01	0.17	0.01	0.01	100.48
sd	0.03	0.01		0.02	0.02	0.02	0.16	0.06	0.1	0.04	0.01	0.08	0.01	0.03	
**CB-PG-3366**	0.01	b.d.l.	b.d.l.	b.d.l.	b.d.l.	0.02	99.77	0.09	0.17	0.07	0.03	0.13	0.01	0.06	100.36
sd	0.01				0.01	0.02	0.26	0.06	0.04	0.08	0.01	0.06	0.02	0.06	
**CB-PGPi-639**	b.d.l.	b.d.l.	0.01	b.d.l.	0.02	0.01	100	0.08	0.16	0.02	0.03	0.09	b.d.l.	0.04	100.51
sd			0.01		0.03	0.02	0.29	0.05	0.04	0.03	0.01	0.05		0.05	
**CB-PT-778**	0.01	0.01	b.d.l.	0.07	0.01	0.02	100.1	0.10	0.03	0.09	0.02	0.05	0.01	0.02	100.48
sd	0.02	0.01		0.18	0.01	0.03	0.55	0.10	0.03	0.08	0.02	0.05	0.02	0.02	
**CB-UMB-3080**	0.02	b.d.l.	0.02	0.01	b.d.l.	0.01	97.81	0.13	1.9	0.04	0.03	0.01	b.d.l.	b.d.l.	99.99
sd	0.02		0.02	0.02		0.01	0.31	0.02	0.19	0.03	0.03	0.02			
**Cl-Pa-Ax**	0.02	0.01	0.01	b.d.l.	0.01	0.02	100.1	0.07	0.05	0.1	0.02	0.01	0.03	0.01	100.43
sd	0.03	0.01	0.01		0.02	0.02	0.4	0.06	0.03	0.07	0.02	0.01	0.04	0.03	
**Cl-PaAx40**	0.01	b.d.l.	b.d.l.	0.01	0.01	0.02	99.34	0.07	0.03	0.17	0.01	0.02	b.d.l.	0.01	99.71
sd	0.01			0.01	0.01	0.02	0.45	0.03	0.07	0.11	0.01	0.04		0.01	
**Fla-Ax**	b.d.l.	b.d.l.	b.d.l.	0.02	0.01	b.d.l.	100.2	0.06	0.04	0.02	0.03	0.05	0.03	0.03	100.51
sd				0.01	0.02		0.40	0.05	0.03	0.03	0.02	0.05	0.02	0.06	
**Hir-Ax**	0.01	0.01	b.d.l.	0.01	0.01	0.01	98.49	b.d.l.	1.93	0.02	0.02	b.d.l.	0.01	b.d.l.	100.52
sd	0.01	0.01		0.01	0.01	0.02	0.40		0.21	0.03	0.02		0.02		
**Is-1507-L**	0.02	b.d.l.	0.01	0.01	b.d.l.	0.01	100	0.13	0.03	0.01	0.03	0.01	0.02	0.04	100.37
sd	0.01		0.02	0.03		0.03	0.31	0.04	0.04	0.02	0.03	0.01	0.03	0.04	
**Is-1904-L**	0.01	b.d.l.	0.01	0.15	b.d.l.	0.03	99.62	0.14	0.27	0.06	0.03	0.05	0.01	0.04	100.42
sd	0.01		0.01	0.15		0.04	0.32	0.07	0.02	0.03	0.03	0.05	0.02	0.05	
**Is-2279-L**	0.01	0.01	0.01	b.d.l.	0.02	0.01	99.85	0.09	0.27	0.05	0.01	0.14	0.02	0.01	100.52
sd	0.02	0.01	0.01		0.02	0.03	0.35	0.12	0.02	0.01	0.01	0.10	0.02	0.02	
**KBG-Ax**	0.03	0.01	0.02	0.02	b.d.l.	0.02	99.00	0.12	b.d.l.	0.29	0.03	0.04	0.02	0.08	99.66
sd	0.01	0.01	0.02	0.03		0.02	0.45	0.11		0.05	0.03	0.05	0.04	0.09	
**Kla-Ax**	0.02	b.d.l.	0.01	0.01	0.01	0.02	97.19	0.05	1.13	1.88	b.d.l.	0.01	0.03	0.03	100.38
sd	0.01		0.01	0.01	0.02	0.05	0.85	0.03	0.15	0.45			0.04	0.07	
**Kol-Ax**	0.01	0.01	0.01	b.d.l.	b.d.l.	b.d.l.	96.43	b.d.l.	3.66	0.03	0.01	0.01	0.02	b.d.l.	100.19
sd	0.02	0.01	0.01				0.49		0.37	0.04	0.02	0.02	0.04		
**Kr-Ax**	0.02	0.01	b.d.l.	0.01	b.d.l.	0.01	100.2	b.d.l.	0.07	0.01	b.d.l.	b.d.l.	b.d.l.	b.d.l.	100.35
sd	0.02	0.01		0.01		0.01	0.3		0.03	0.01					

**Lag-Ax**	0.01	0.01	b.d.l.	0.01	0.01	b.d.l.	100.2	0.04	0.03	0.06	0.03	0.02	0.03	0.05	100.48
sd	0.01	0.01		0.02	0.01		0.15	0.05	0.03	0.05	0.03	0.02	0.02	0.06	
**Lan-Ax**	0.04	0.01	b.d.l.	0.02	0.01	0.01	99.64	0.09	0.15	0.24	0.01	0.03	0.02	b.d.l.	100.26
sd	0.02	0.01		0.02	0.02	0.01	0.20	0.07	0.04	0.05	0.02	0.04	0.03		
**Lon-Ax**	0.03	b.d.l.	0.01	0.01	0.02	0.93	99.14	0.03	0.03	0.03	0.02	b.d.l.	0.02	b.d.l.	100.58
sd	0.07		0.02	0.02	0.03	0.26	0.56	0.05	0.03	0.04	0.02		0.01		
**ME-LS-Ax**	0.18	0.01	0.01	b.d.l.	b.d.l.	0.02	100.4	0.07	0.01	0.01	0.02	0.04	0.01	b.d.l.	100.8
sd	0.03	0.01	0.02			0.01	0.43	0.05	0.01	0.02	0.01	0.05	0.01		
**PK-04-L**	0.01	b.d.l.	0.01	0.01	0.01	0.01	100.1	0.13	0.02	0.25	0.02	0.02	0.03	0.02	100.61
sd	0.01		0.01	0.02	0.01	0.01	0.39	0.04	0.02	0.04	0.02	0.05	0.05	0.02	
**PK-06-L**	0.01	0.02	0.01	0.01	b.d.l.	0.01	100.0	b.d.l.	0.05	0.2	0.01	0.04	0.02	0.07	100.46
sd	0.01	0.01	0.01	0.01		0.01	0.41		0.02	0.02	0.01	0.02	0.04	0.12	
**PK-51-Ax**	0.02	b.d.l.	0.01	b.d.l.	0.01	0.01	99.87	0.02	0.10	0.17	b.d.l.	0.03	0.10	b.d.l.	100.35
sd	0.01		0.01		0.01	0.01	0.24	0.02	0.06	0.09		0.03	0.08		
**PK-52-Ax**	0.01	b.d.l.	b.d.l.	0.01	0.05	0.03	99.53	0.39	0.09	0.16	0.03	0.01	0.13	0.02	100.47
sd	0.01			0.01	0.02	0.01	0.48	0.04	0.07	0.02	0.02	0.01	0.12	0.05	
**PK-66-Ax**	0.03	b.d.l.	0.01	0.02	0.06	0.01	99.35	0.51	0.06	0.14	0.01	0.06	0.11	0.06	100.43
sd	0.01		0.01	0.01	0.02	0.01	0.46	0.04	0.10	0.06	0.03	0.03	0.08	0.11	
**PK-67-Ax**	0.07	b.d.l.	b.d.l.	0.01	0.01	0.02	99.48	b.d.l.	0.12	0.26	0.02	0.07	0.16	0.02	100.24
sd	0.08			0.01	0.01	0.02	0.90		0.11	0.24	0.02	0.11	0.15	0.05	
**PK-Or**	0.01	b.d.l.	0.01	0.02	0.01	0.03	99.88	0.18	0.01	0.24	0.01	0.06	0.03	0.04	100.53
sd	0.01		0.01	0.03	0.01	0.04	0.32	0.05	0.01	0.01	0.01	0.04	0.04	0.03	
**PK-Pe**	b.d.l.	b.d.l.	0.01	0.03	0.01	0.03	100.2	0.07	0.14	0.05	0.01	0.03	0.04	0.02	100.58
sd			0.01	0.03	0.02	0.06	0.41	0.04	0.07	0.02	0.01	0.02	0.05	0.03	
**PK-Pg**	0.01	0.01	0.01	0.02	0.02	0.11	98.63	0.01	1.13	0.13	0.02	0.43	0.1	0.01	100.62
sd	0.01	0.01	0.01	0.02	0.01	0.04	0.43	0.01	0.20	0.02	0.01	0.06	0.07	0.02	
**PK-Sp**	0.02	b.d.l.	b.d.l.	b.d.l.	0.02	0.02	99.93	0.05	0.06	0.28	0.04	0.03	0.04	0.02	100.51
sd	0.02			0.01	0.02	0.02	0.24	0.05	0.04	0.03	0.02	0.05	0.06	0.04	
**PsP-Ax**	b.d.l.	b.d.l.	0.01	0.01	0.02	b.d.l.	99.09	0.08	0.22	0.21	0.02	0.23	b.d.l.	b.d.l.	100.82
sd			0.02	0.01	0.02		0.47	0.09	0.09	0.13	0.02	0.38			
**Rem-Ax102**	0.03	0.01	b.d.l.	0.01	b.d.l.	0.01	99.12	n.a.	0.13	0.13	b.d.l.	b.d.l.	b.d.l.	b.d.l.	99.45
sd	0.02	0.01		0.01		0.01	0.40		0.09	0.06					
**Rem-Ax4**	0.2	0.01	0.01	0.01	0.01	0.13	99.57	n.a.	0.28	0.05	0.01	0.01	b.d.l.	0.07	100.37
sd	0.07	0.01	0.01	0.01	0.01	0.02	0.21		0.07	0.02	0.02	0.02		0.09	
**Rem-Ax62**	0.11	0.01	b.d.l.	0.02	0.01	0.03	99.95	0.12	0.16	0.13	0.03	0.01	0.07	b.d.l.	100.67
sd	0.01	0.02		0.03	0.01	0.03	0.27	0.10	0.07	0.14	0.04	0.03	0.05		
**Rem-Ax78**	0.01	0.01	b.d.l.	b.d.l.	b.d.l.	0.02	100.1	n.a.	0.01	0.08	0.01	b.d.l.	b.d.l.	b.d.l.	100.25
sd	0.01	0.01				0.02	0.86		0.02	0.03	0.01				
**SBV-940**	0.65	b.d.l.	0.01	0.01	0.01	0.04	97.28	0.1	1.58	0.14	0.02	0.55	0.01	0.01	100.41
sd	0.11		0.01	0.01	0.01	0.04	0.76	0.08	0.32	0.04	0.04	0.10	0.01	0.01	
**SPEz-Ax**	0.03	b.d.l.	0.01	0.01	0.01	0.01	98.54	0.15	1.25	0.09	0.02	0.02	0.01	b.d.l.	100.17
sd	0.03		0.02	0.01	0.03	0.03	0.74	0.10	0.43	0.06	0.03	0.01	0.02		
**Ver-Ax**	0.01	b.d.l.	0.02	0.01	0.01	0.01	98.09	0.06	0.32	1.3	0.01	0.61	0.01	0.05	100.48
sd	0.01		0.03	0.02	0.01	0.01	0.29	0.03	0.08	0.10	0.02	0.02	0.01	0.05	
**Vil-Ax**	0.02	b.d.l.	0.01	0.01	0.01	0.01	99.64	0.08	0.08	0.18	0.03	0.04	0.11	0.07	100.28
sd	0.01		0.01	0.01	0.01	0.01	0.25	0.08	0.08	0.07	0.04	0.04	0.08	0.08	

Supplementary Table 4

Measured lead isotope ratios for all samples. *The relatively high estimated standard deviations are due to the very low lead content in the sample.

Sample	Provenienza	$^{206}Pb/^{204}Pb$	2σ	$^{207}Pb/^{204}Pb$	2σ	$^{208}Pb/^{204}Pb$	2σ	$^{207}Pb/^{206}Pb$	2σ	$^{208}Pb/^{206}Pb$	2σ
**Aq-Ax**	Aquileia	17.9179	0.0005	15.6424	0.0006	38.1285	0.0016	0.8730	0.0001	2.1280	0.0001
**Arc-Ax**	Arcugnano/Valle Fontega	18.2649	0.0007	15.6743	0.0008	38.4844	0.0023	0.8582	0.0002	2.1071	0.0001
**BL-Ax76**	Santorso/Bocca Lorenza	18.5064	0.0009	15.6709	0.0008	38.6621	0.0023	0.8468	0.0001	2.0891	0.0001
**BL-Ax77**	Santorso/Bocca Lorenza	18.5334	0.0015	15.6627	0.0014	38.6239	0.0032	0.8451	0.0002	2.0841	0.0001
**BL-Ax93**	Santorso/Bocca Lorenza	18.5371	0.0012	15.6464	0.0010	38.5326	0.0028	0.8441	0.0001	2.0787	0.0001
**Camp-Ax** *	Campegine (RE)	18.2121	0.2241	15.5971	0.1939	38.2415	0.4317	0.8564	0.0105	2.0987	0.0234
**Canz-Ax**	San Canziano	18.5104	0.0010	15.6710	0.0010	38.6817	0.0034	0.8466	0.0001	2.0898	0.0001
**CB-AsSM-6984**	Assisi/San Martino	18.7180	0.0008	15.6985	0.0007	39.0229	0.0023	0.8387	0.0001	2.0848	0.0001
**CB-CdC-3770**	Città di Castello	18.6040	0.0009	15.6836	0.0008	38.8128	0.0026	0.8430	0.0001	2.0862	0.0001
**CB-MaBa-188**	Marsciano/ Badiola	18.3396	0.0005	15.6549	0.0005	38.4561	0.0014	0.8536	0.0001	2.0969	0.0001
**CB-OLM-842**	Olmeto	18.7976	0.0010	15.7114	0.0009	39.0842	0.0025	0.8358	0.0001	2.0792	0.0001
**CB-PG-3366**	Perugia	18.6927	0.0006	15.6988	0.0006	38.9780	0.0017	0.8398	0.0001	2.0852	0.0001
**CB-PGPi-639**	Perugia/Pila	18.7806	0.0008	15.7060	0.0009	39.0601	0.0029	0.8363	0.0001	2.0798	0.0001
**CB-PT-778**	Perugia territorio (PG)	18.7033	0.0007	15.6867	0.0006	38.9109	0.0017	0.8387	0.0001	2.0804	0.0001
**CB-UMB-3080**	Umbertide	18.6217	0.0012	15.6771	0.001	38.7949	0.0026	0.8419	0.0001	2.0833	0.0001
**Cl-Pa-Ax***	Celletta dei Passeri (Forlì)	18.7653	0.0138	15.7070	0.0115	39.0287	0.0275	0.8370	0.0001	2.0796	0.0002
**CL-Pa-Ax40**	Celletta dei Passeri, Forlì	18.7702	0.0013	15.7118	0.0008	39.0326	0.0029	0.8371	0.0001	2.0795	0.0001
**Dam-Ax**	Dambel	18.6324	0.0007	15.6675	0.0007	38.7955	0.0020	0.8409	0.0001	2.0821	0.0001
**Fla-Ax**	Flavon	18.0054	0.0012	15.6477	0.0015	38.2097	0.0052	0.8691	0.0001	2.1221	0.0001
**Hir-Ax**	Nova Levante-Welschnofen, Hirzlsteig (BZ)	18.4816	0.0020	15.5890	0.0018	38.3374	0.0056	0.8435	0.0001	2.0743	0.0002
**Is-1507-L**	Isera La Torretta	18.2873	0.0040	15.6342	0.0032	38.3281	0.0107	0.8549	0.0001	2.0958	0.0004
**Is-1904-L**	Isera La Torretta	18.8076	0.0008	15.7062	0.0008	39.0743	0.003	0.8351	0.0001	2.0775	0.0001
**Is-2279-L***	Isera La Torretta	18.4152	0.3784	15.6555	0.3228	38.3633	0.7866	0.8501	0.0164	2.0836	0.0399
**KBG-Ax**	Kanzianiberg	18.4942	0.0007	15.6632	0.0007	38.6180	0.0021	0.8469	0.0001	2.0881	0.0001
**Kla-Ax**	Gurnitz	18.4881	0.0010	15.6655	0.0011	38.6327	0.0035	0.8473	0.0001	2.0897	0.0001
**Kol-Ax**	Barbiano-Barbian, Kollmann-Colma (BZ)	18.4986	0.0017	15.6022	0.0015	38.4461	0.0036	0.8434	0.0001	2.0783	0.0001
**Kr-Ax**	Castelrotto-Kastelruth,	18.8555	0.0012	15.7013	0.0012	38.9012	0.0035	0.8327	0.0001	2.0632	0.0001

	Gamertinerhof (BZ)										
**Lag-Ax**	Lagolo	18.0884	0.0009	15.6678	0.0010	38.3523	0.0033	0.8662	0.0001	2.1203	0.0001
**Lan-Ax**	Lana, Gaulschlucht (BZ)	18.5097	0.0015	15.6205	0.0014	38.5150	0.0040	0.8439	0.0001	2.0807	0.0001
**Lon-Ax***	Lonato (BS)	18.7440	0.0416	15.8493	0.0319	38.9512	0.0794	0.8462	0.0003	2.0797	0.0006
**ME-LS-Ax**	Montecchio Emilia, La sacca (RE)	18.3123	0.0071	15.6478	0.0060	38.3766	0.0146	0.8545	0.0001	2.0957	0.0001
**PK-04-L**	Vadena-Pfatten/Pigloner Kopf Abri (BZ)	18.2737	0.0011	15.6809	0.0012	38.5029	0.0041	0.8581	0.0001	2.1070	0.0001
**PK-06-L**	Vadena-Pfatten, Pigloner Kopf (BZ)	18.2638	0.0010	15.6742	0.0010	38.4336	0.0027	0.8582	0.0001	2.1043	0.0001
**PK-51-Ax**	Vadena-Pfatten, Pigloner Kopf (BZ)	18.2619	0.0013	15.6782	0.0012	38.4940	0.0034	0.8585	0.0001	2.1079	0.0001
**PK-52-Ax**	Vadena-Pfatten, Pigloner Kopf (BZ)	18.2649	0.0014	15.6787	0.0017	38.4964	0.0049	0.8584	0.0001	2.1076	0.0001
**PK-66-Ax**	Vadena-Pfatten, Pigloner Kopf (BZ)	18.2624	0.0010	15.6781	0.0012	38.4931	0.0033	0.8585	0.0001	2.1078	0.0001
**PK-67-Ax**	Vadena-Pfatten, Pigloner Kopf (BZ)	18.2673	0.0008	15.6766	0.0009	38.4947	0.0028	0.8582	0.0001	2.1073	0.0001
**PK-Or**	Vadena-Pfatten/Pigloner Kopf Abri (BZ)	18.2274	0.0033	15.6376	0.0042	38.3806	0.0146	0.8579	0.0001	2.1057	0.0004
**PK-Pe**	Vadena-Pfatten/Pigloner Kopf Abri (BZ)	18.2481	0.0014	15.6265	0.0013	38.4076	0.0036	0.8563	0.0001	2.1047	0.0001
**PK-Pg**	Vadena-Pfatten/Pigloner Kopf Abri (BZ)	18.2929	0.0010	15.6545	0.0009	38.4871	0.0019	0.8558	0.0001	2.1039	0.0001
**PK-Sp**	Vadena-Pfatten/Pigloner Kopf Abri (BZ)	18.2798	0.0013	15.6795	0.0015	38.5080	0.0049	0.8578	0.0001	2.1066	0.0001
**PsP-Ax**	Ponte San Pietro	18.5716	0.0027	15.5975	0.0033	38.5795	0.0110	0.8399	0.0001	2.0774	0.0003
**Rem-Ax102**	Remedello (BS)	18.7742	0.0007	15.7003	0.0008	38.9650	0.0023	0.8363	0.0001	2.0754	0.0001
**Rem-Ax4**	Remedello (BS)	18.6255	0.0020	15.6816	0.0017	38.7824	0.0038	0.842	0.0001	2.0823	0.0001
**Rem-Ax62**	Remedello (BS)	18.6895	0.0016	15.6952	0.0015	38.9091	0.0042	0.8398	0.0001	2.0818	0.0001
**Rem-Ax78**	Remedello (BS)	18.7221	0.0033	15.6837	0.0027	38.8850	0.0065	0.8377	0.0001	2.0770	0.0001
**SBV-940**	Casanuova di san Biagio della Valle (Marsciano, Perugia)	18.5822	0.0005	15.6876	0.0005	38.8298	0.0015	0.8442	0.0001	2.0897	0.0001
**SPEz-Ax**	San Polo d'Enza (RE)	18.5230	0.0067	15.6548	0.0057	38.6436	0.0152	0.8452	0.0001	2.0865	0.0002
**Ver-Ax**	Vervò (TN)	18.3817	0.0008	15.6614	0.0008	38.5413	0.0022	0.8520	0.0001	2.0967	0.0001
**Vil-Ax**	Fresach	18.2663	0.0009	15.6752	0.0011	38.4952	0.0038	0.8582	0.0001	2.1075	0.0001

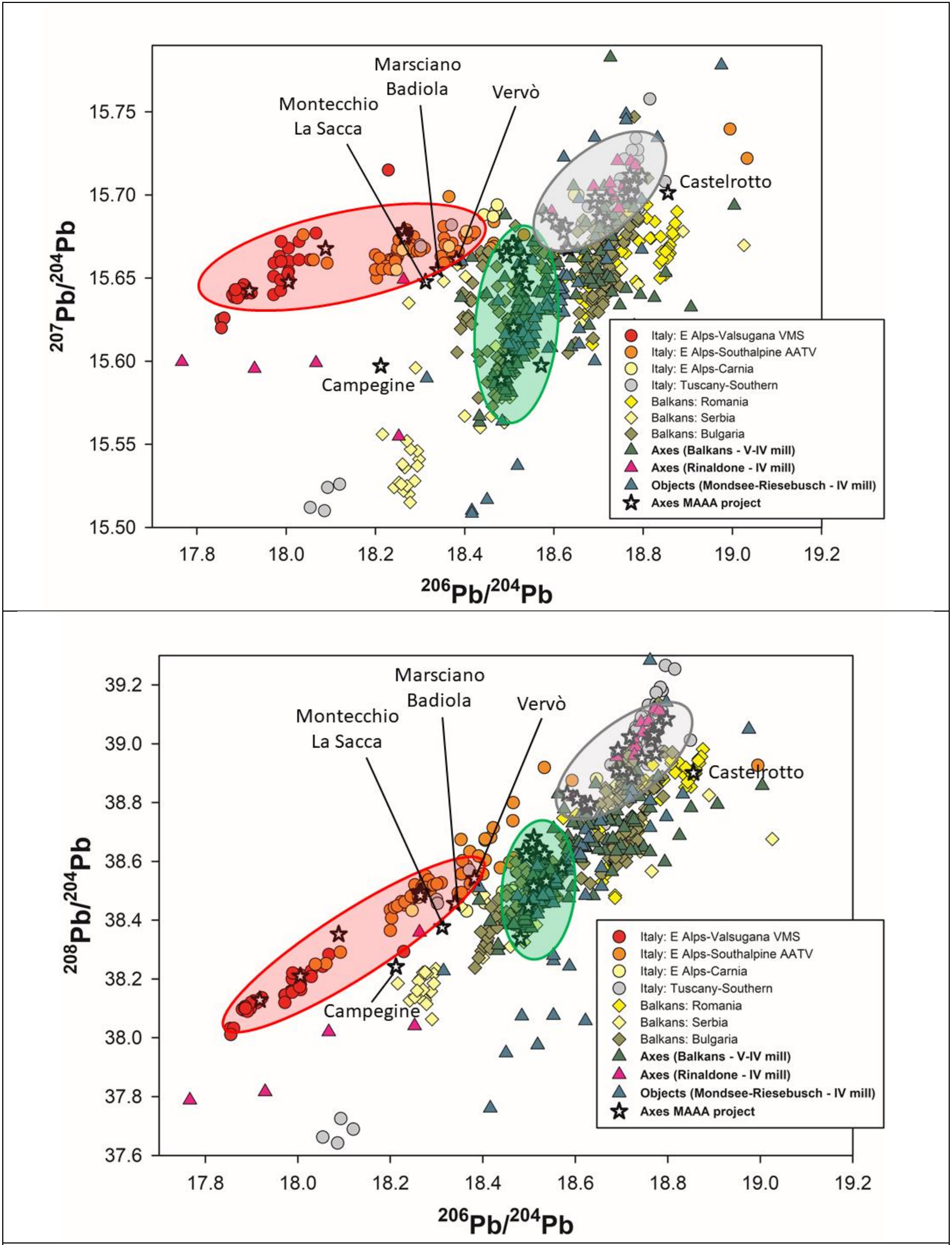

Supplementary Fig. 1. 2D projections of the 3D isotopic space for the measured copper axes as in Fig. 1, compared to the circulating objects in the 5th and 4th millenniums BC[9–12].

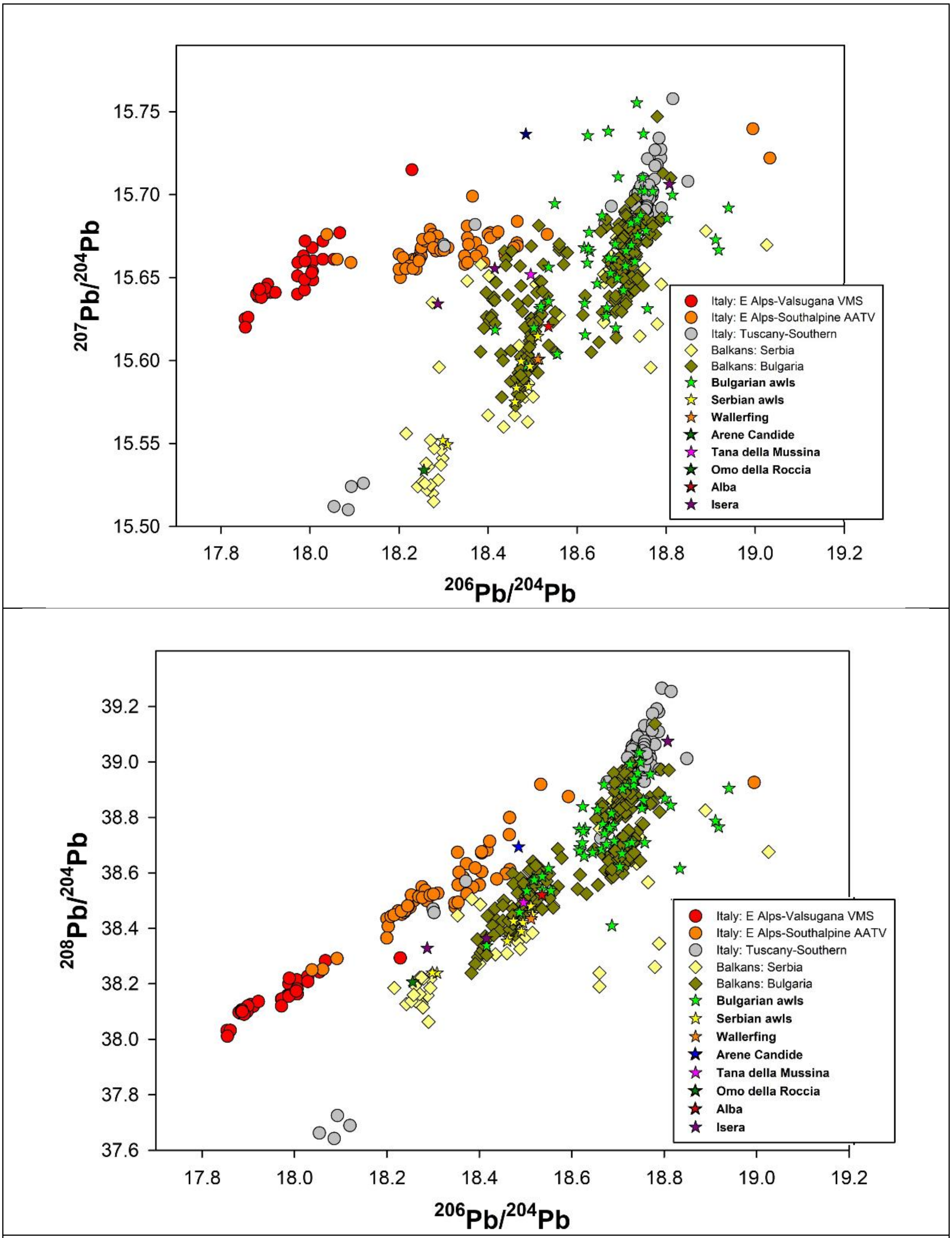

Supplementary Fig. 2. 2D projections of the 3D isotopic space for the measured Late Neolithic awls as in Fig. 1 of ref. 13. The original figure has been revised by adding the awl LIA data reported after 2020: Isera (present work) and Omo della Roccia[14].

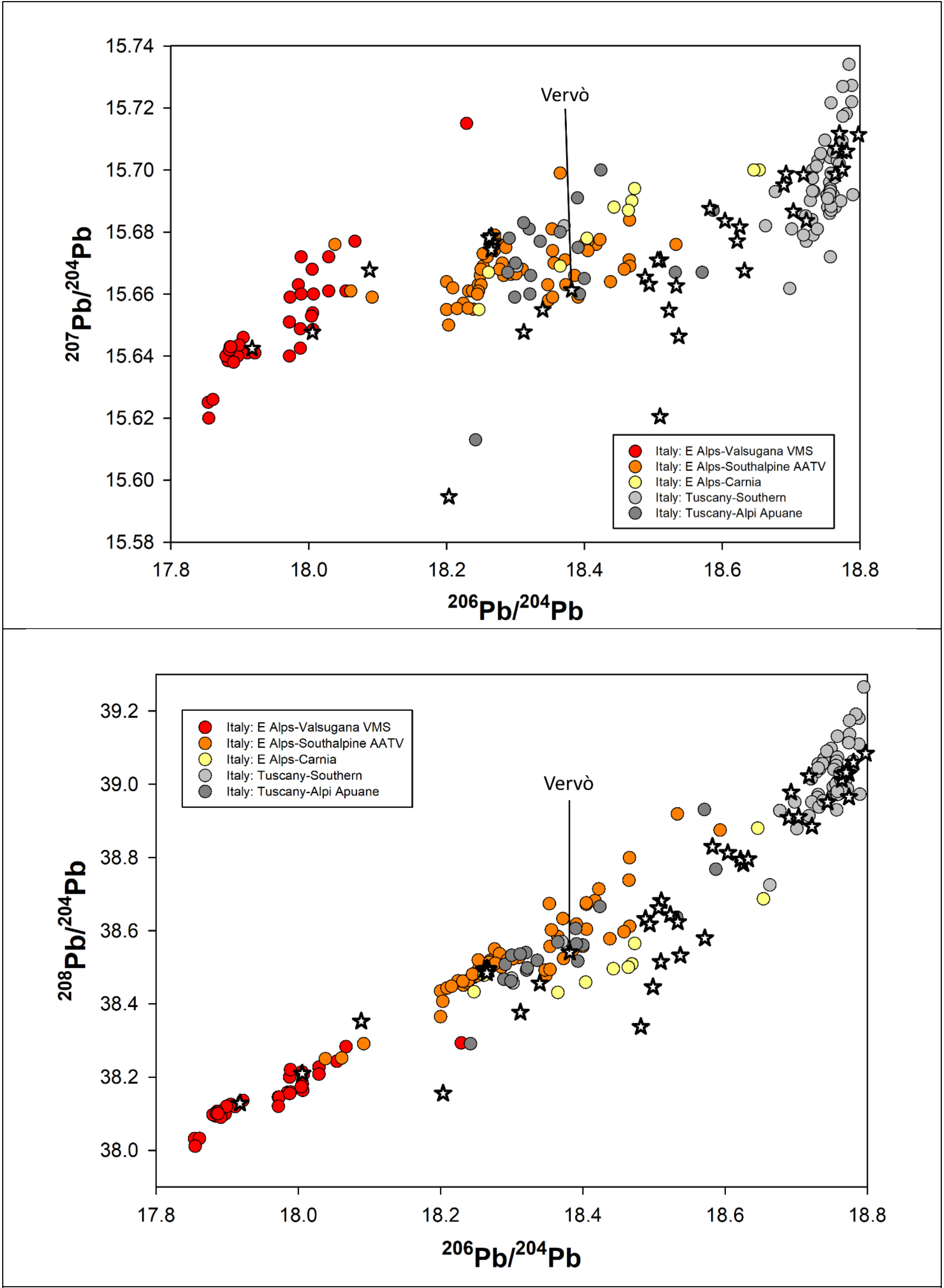

Supplementary Fig. 3. 2D projections of the 3D isotopic space for the measured copper axes as in Fig. 1. The LI data of the Vervò axe are at the boundary of the Alpine ore field, and they are compatible with the ores of Northern Tuscany (Apuanian Alps).

Supplementary Material References

1. Villa, I. M. Lead isotopic measurements in archeological objects. *Archaeol Anthropol Sci* **1**, 149–153 (2009).

2. White, W. M., Albarède, F. & Télouk, P. High-precision analysis of Pb isotope ratios by multi-collector ICP-MS. *Chem Geol* **167**, 257–270 (2000).

3. Rehkämper, M. & Mezger, K. Investigation of matrix effects for Pb isotope ratio measurements by multiple collector ICP-MS: verification and application of optimized analytical protocols. *J. Anal. At. Spectrom.* **15**, 1451–1460 (2000).

4. De Marinis, R. C. & Pedrotti, A. L'età del Rame nel versante italiano delle Alpi centro-occidentali. In *Atti della XXXI Riunione Scientifica dell'IIPP, Courmayeur, 1994* 247–300 (1997).

5. *Roma prima del mito: Abitati e necropoli dal neolitico alla prima eta'dei metalli nel territorio di roma (VI-III millennio aC).* (Archaeopress Publishing Ltd., 2020).

6. Valzogher, E. Cronologia assoluta dei pugnali tipo Remedello e Spilamberto. in *In Le manifestazioni del sacro e l'Età del Rame nella regione alpina e nella pianura padana.* (ed. De Marinis, R. C.) 239–258 (Euro Team, 2016).

7. Miari, M., Bestetti, F. & Rasia, P. A. La necropoli eneolitica di Celletta dei Passeri (Forlì): analisi delle sepolture e dei corredi funerari. *Rivista di scienze preistoriche* **LXVII**, 145–208 (2017).

8. Pedrotti, A. *et al.* The Bell Beaker Rock Sanctuary Pigloner Kopf (South Tyrol, Italy): Burnt Offerings and Local Metallurgy in the Eastern Alps. in *The Bell Beaker Culture in All Its Forms: Proceedings of the 22nd Meeting of 'Archéologie et Gobelets' 2021 (Geneva, Switzerland)* 265 (Archaeopress Publishing Ltd., 2022).

9. Klassen, L. & Stürup, S. Decoding the Riesebusch-copper:Lead-Isotope Analysis applied to Early Neolithic Copper Finds from South Scandinavia. *Praehistorische Zeitschrift* **76**, (2001).

10. Dolfini, A., Angelini, I. & Artioli, G. Copper to Tuscany – Coals to Newcastle? The dynamics of metalwork exchange in early Italy. *PLoS One* **15**, (2020).

11. Pernicka, E., Begemann, F., Schmitt-Strecker, S. & Wagner, G. A. Eneolithic and Early Bronze Age copper artefacts from the Balkans and their relation to Serbian copper ores. *Praehstorische Zeitschrift* **68**, 1–57 (1993).

12. Gale, N. H. *et al.* Early metallurgy in Bulgaria. in *Godishnik Nov Bulgarski Universitet IV-V* 102–68 (2000).

13. Artioli, G., Canovaro, C., Nimis, P. & Angelini, I. LIA of Prehistoric Metals in the Central Mediterranean Area: A Review. *Archaeometry* **62**, Suppl. 1, 53–85 (2020).

14. Angelini, I., Canovaro, C., Artioli, G. & Migliavacca, M. Uomo della roccia: la lesina di rame. in Il sito di Uomo della Roccia (Muzzolon di Cornedo Vicentino) - Comunità e ambiente prealpino dal quinto millennio a.C. (ed. Migliavacca, M.) 77-84 (SAP Società Archeologica s.r.l., 2020).
